# Inhibition of parasite invasion by monoclonal antibody against epidermal growth factor-like domain of *Plasmodium vivax* merozoite surface protein 1 paralog

**DOI:** 10.1038/s41598-019-40321-2

**Published:** 2019-03-07

**Authors:** Jin-Hee Han, Yang Cheng, Fauzi Muh, Md Atique Ahmed, Jee-Sun Cho, Myat Htut Nyunt, Hye-Yoon Jeon, Kwon-Soo Ha, Sunghun Na, Won Sun Park, Seok-Ho Hong, Ho-Joon Shin, Bruce Russell, Eun-Taek Han

**Affiliations:** 1 Department of Medical Environmental Biology and Tropical Medicine, School of Medicine, Kangwon National University, Chuncheon, Gangwon-do Republic of Korea; 2 Department of Microbiology and Immunology, University of Otago, Dunedin, 9054 New Zealand; 3 Department of Public Health and Preventive Medicine, Laboratory of Pathogen Infection and Immunity, Wuxi School of Medicine, Jiangnan University, Wuxi, Jiangsu People’s Republic of China; 4 Department of Microbiology, Yong Loo Lin School of Medicine, National University of Singapore, National University Health System, Singapore 117597, Singapore; Singapore Immunology Network (SIgN), A*STAR, Singapore, 138648 Singapore; 5 Jenner Institute Laboratories, Old Road Campus Research Building, University of Oxford, Oxford, United Kingdom; 6 Department of Medical Research, Yangon, Myanmar; 7 Department of Cellular and Molecular Biology, School of Medicine, Kangwon National University, Chuncheon, Gangwon-do Republic of Korea; 8 Department of Obstetrics and Gynecology, School of Medicine, Kangwon National University, Chuncheon, Gangwon-do Republic of Korea; 9 Department of Physiology, School of Medicine, Kangwon National University, Chuncheon, Gangwon-do Republic of Korea; 10 Department of Internal Medicine, School of Medicine, Kangwon national University, Chuncheon, Gangwon-do Republic of Korea; 11 Department of Microbiology, Ajou University School of Medicine, and Department of Biomedical Science, Ajou University Graduate School of Medicine, Suwon, Gyeonggi-do Republic of Korea

## Abstract

The *Plasmodium vivax* merozoite surface protein 1 paralog (PvMSP1P), which has epidermal growth factor (EGF)-like domains, was identified as a novel erythrocyte adhesive molecule. This EGF-like domain (PvMSP1P-19) elicited high level of acquired immune response in patients. Antibodies against PvMSP1P significantly reduced erythrocyte adhesion activity to its unknown receptor. To determine PvMSP1P-19-specific antibody function and B-cell epitopes in vivax patients, five monoclonal antibodies (mAbs) and 18-mer peptides were generated. The mAb functions were determined by erythrocyte-binding inhibition assay and invasion inhibition assay with *P. knowlesi*. B-cell epitopes of PvMSP1P-19 domains were evaluated by peptide microarray. The *pvmsp1p-19* sequences showed limited polymorphism in *P. vivax* worldwide isolates. The 1BH9-A10 showed erythrocyte binding inhibitory by interaction with the N-terminus of PvMSP1P-19, while this mAb failed to recognize PkMSP1P-19 suggesting the species-specific for *P. vivax*. Other mAbs showed cross-reactivity with PkMSP1P-19. Among them, the 2AF4-A2 and 2AF4-A6 mAb significantly reduced parasite invasion through C-terminal recognition. The linear B-cell epitope in naturally exposed *P. vivax* patient was identified at three linear epitopes. In this study, PvMSP1P-19 N-terminal-specific 1BH9-A10 and C-terminal-specific 2AF4 mAbs showed functional activity for epitope recognition suggesting that PvMSP1P may be useful for vaccine development strategy for specific single epitope to prevent *P. vivax* invasion.

## Introduction

Among the five *Plasmodium* species that cause malaria in humans, *P. vivax* is the most widely distributed and causes infections worldwide outside Sub-Saharan African regions^1^. A vaccine to protect against *P. vivax* is especially needed due to widespread drug resistance in some countries. However, *P. vivax* blood-stage vaccine development has been limited because of a lack of understanding of invasion mechanisms^2^. Identifying individual antigen and/or antibody functions is one alternative approach to vaccine development.

Many merozoite surface antigens have been discovered to be highly immunogenic in patients who are naturally exposed to human invasive malaria parasites^3,4^. Likewise, *P. vivax* merozoite surface protein 1 (PvMSP1) is currently suggested as one of the most advanced vaccine candidates in the vivax parasite blood stage^5,6^. The merozoite surface antigens come up as a critical role at initial contact by complex form of merozoite surface antigen with host cells and immune evasion during merozoite internalization by shedding of the surface coat^7,8^. Updating knowledge figures out PfMSP1 processing and functions are important for parasite egress from red blood cells^9^. Although merozoite surface antigens showed immune evasion activity, it could be easier to target by the host antibody than apical organelle antigens because it is easily exposed to the host immune system^10,11^. In contrast, apical organelles are only exposed to the immune system for short periods compared to surface molecules due to the rapid invasion process. Hence, various merozoite antigens have been proposed as a potential vaccine candidate, not only MSP1 but also other surface antigens^5^. In particular, glycosylphosphatidylinositol (GPI)-anchored merozoite surface antigens, including MSP2, MSP4, MSP5, MSP8, and MSP10, were considered as novel blood-stage vaccine candidates^5,10,12–14^. However, these antigens have a critical disadvantage for vaccine development because of high polymorphism. The C-terminal fragments of PvMSP1, PvMSP1P, PvMSP8, and PvMSP10 contain identical cysteine residues within two of the epidermal growth factor (EGF)-like domains^15^, which was confirmed by conformational crystal structures in various *Plasmodium* spp.^16–18^.

Recently, the novel antigen PvMSP1P was reported to localize on the merozoite surface by a GPI-anchored motif^19^. This antigen led to erythrocyte adhesion by two EGF-like domains (PvMSP1P-19) at the C-terminus and showed a high level of acquired immune responses in vivax patients^19,20^. The functional antibody against PvMSP1P-19 from a vivax patient demonstrated inhibition activities for erythrocyte adhesion^19,20^. PvMSP1P induced predominant IgG1 and IgG3 antibody responses in vivax-infected patients^21,22^. These two IgG isotypes are highly induced by both of antibody-dependent cellular cytotoxicity (ADCC) and complement-dependent cytotoxicity (CDC) effect. Likewise, antibodies against PfMSP1-19 induced IgG1 and IgG3 and showed merozoite invasion blocking activity by interruption of processing^23^. The cellular immune response properties in mice showed that Th1 cytokine levels were significantly higher than those in PvMSP1-19 immunized mice. Likewise, PvMSP1P-19 strongly induced a specific cellular immune response by activation of IFN-γ-producing effector cells in natural human infections^21^. These findings might reflect that PvMSP1P is a feasible vivax vaccine candidate.

A high priority of the invasion blocking vaccine discovery for the blood stage is to identify specific antibody functions and immune properties in patients. In the present study, we have demonstrated the functional epitope for inhibition of erythrocyte binding and parasite invasion by monoclonal antibodies (mAbs). The result will provide an understanding of the protection against *P. viv*ax invasion into erythrocytes and deserve consideration for vaccine development.

## Results

### Primary structure and sequence diversity of PvMSP1P-19

**Figure 1:**
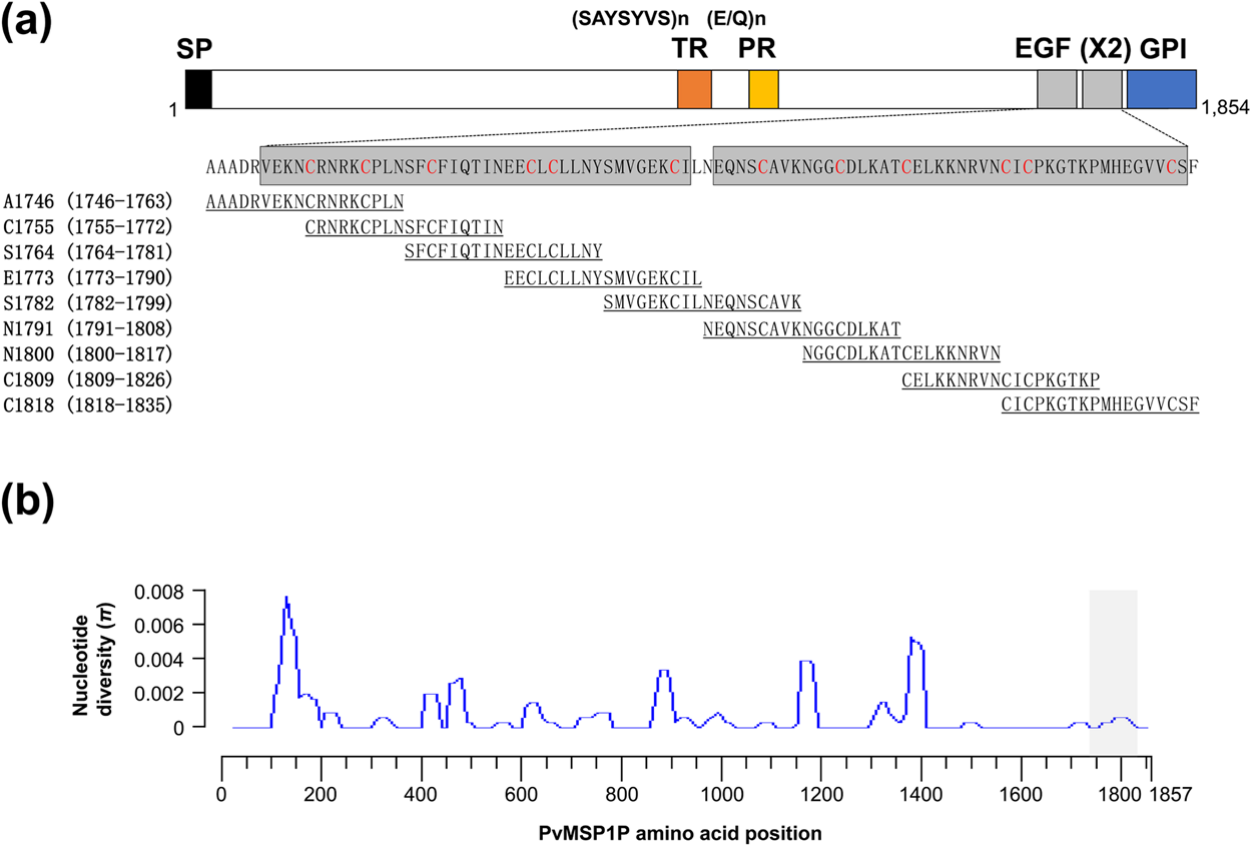
Schematic structure of PvMSP1P-19 and sequence diversity in worldwide isolate. (**a**) The schematic diagram shown PvMSP1P primary structure. The signal peptide (SP, black box), tandem repeat (TR, orange box), polymorphic Glu/Gln-rich region (PR, yellow box), epidermal growth factor like (EGF, Gray box), and the glycosylphosphatidylinositol (GPI, blue box) indicates. The 18-mer peptides indicated with amino acid position and sequence for peptide microarray. (**b**) Sliding window plot showing nucleotide diversity (*π*) values of PvMSP1P using 66 worldwide isolates. The grey box represents two EGF-like domains at amino acid positions 1751 to 1834.

PvMSP1P encoded by 1,854 amino acid (aa.) sequence with a 214.5 kDa molecular weight. The primary structure of PvMSP1P has been predicted to contain signal peptide (SP, 1 to 28 aa.) at N-terminus, two EGF-like domains (EGF, 1749 to 1834 aa.) and GPI anchored domain (GPI, 1834 to 1854 aa.) at C-terminus (Fig. 1a). The central part of ectodomain has hepta-peptide tandem repeat region (TR, 905 to 918 aa.), and Glu/Gln-rich region (PR, 1157 to 1172 aa.) (Fig. 1a).

Sixty-six isolates of *pvmsp1p* from PlasmoDB (http://plasmodb.org/) originating from 10 countries (Brazil, China, Columbia, India, Mauritania, Mexico, North Korea, Peru, Papua New Guinea, and Thailand) were used for nucleotide diversity analysis. The nucleotide diversity (π) showed 0.00066 within worldwide isolates, thus indicating that *pvmsp1p* had limited polymorphism (Fig. 1b). The thirty *pvmsp1p* sequences from Republic of Korea (ROK), Thailand and Myanmar were newly sequenced in this study and sequence alignment indicated a conserved EGF-like domain (Table 1). The sequence alignfment of thirty isolates are described as Supplementary Data 1. The nucleotide diversity (π) comparison between EGF-like domain of *pvmsp1-19* (0.00060) and *pvmsp1p-19* (0.00032) indicated that low polymorphism occurred in *pvmsp1p-19* (Supplementary Fig. S1).

**Table 1:** Vivax patient field isolate information.

	Vivax patients in endemic countries			Non-endemic area
	Republic of Korea	Thailand	Myanmar	Republic of Korea
Total (*n*)	11	7	12	30
Age (year)				
Mean ± S.D.	48.50 ± 30.78	36.80 ± 10.99	22.09 ± 7.78	9.20 ± 2.12
Range	20–89	23–46	14–40	6–13
Parasitaemia (%)				
Mean ± S.D.	0.1813 ± 0.1792	0.1916 ± 0.1089	0.0849 ± 0.0531	—
Range	0.0100–0.5700	0.07504–0.3049	0.0241–0.1964	—

ROK, Republic of Korea; S.D., standard deviation.

### Characterization of monoclonal antibodies

**Figure 2:**
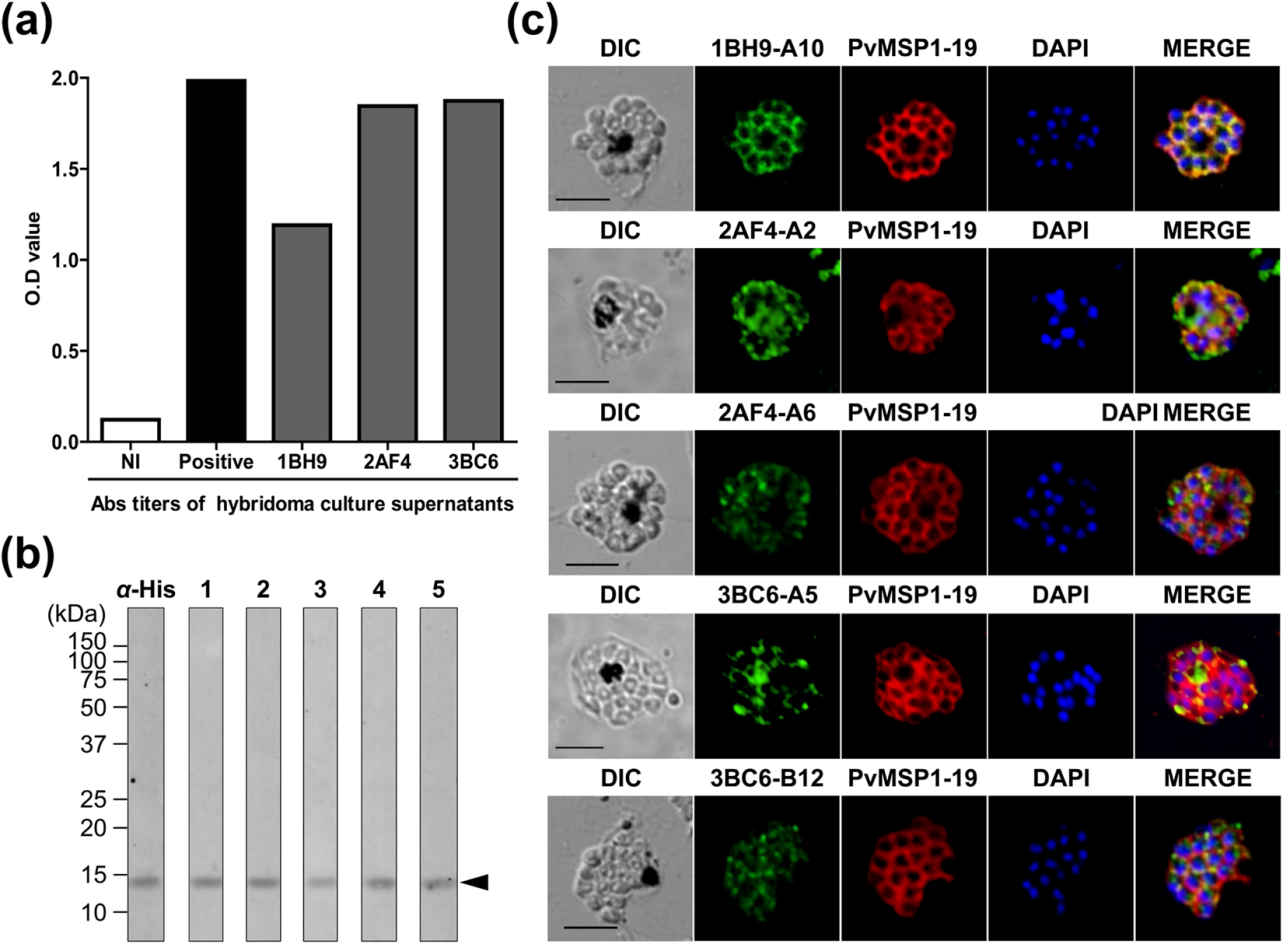
PvMSP1P-19 monoclonal antibody production and validation. (**a**) Three clones were successfully hybridized to produce monoclonal antibodies, and hybridoma culture supernatants were obtained. The OD values were measured by indirect ELISA at 405 nm. Antigen was used at concentrations of 1 µg/ml. (**b**) A western blot showing five monoclonal antibodies reacting with PvMSP1P-19. The approximately 14 kDa specific band indicates rPvMSP1P-19 (arrow head). His, penta-anti-His antibody; lanes 1–5, anti-PvMSP1P-19 monoclonal antibodies as follows: lane 1, 1BH9-A10; lane 2, 2AF4-A2; lane 3, 2AF4-A6; lane 4, 3BC6-A5; and lane 5, 3BC6-B12. (**c**) Reactivity detection for monoclonal antibodies with native PvMSP1P by immunofluorescence assay. The mature schizont of *P. vivax* was dual labelled with PvMSP1P-19 monoclonal antibodies (green) and rabbit immune sera against PvMSP1-19 (red, merozoite surface marker). Nuclei are visualized with DAPI (blue). Bar indicate 5 μm.

To qualify monoclonal antibodies, three hybridoma cells were selected for cloning by limiting dilution based on their high absorbance in ELISA. The hybridoma cells were named 1BH9, 2AF4, and 3BC6 and showed O.D. values of 1.191, 1.845, and 1.873, respectively (Fig. 2a). In total, five mAbs (1BH9-A10: IgG1-κ, 2AF4-A2: IgG3-κ, 2AF4-A6: IgG3-κ, 3BC6-A5: IgG2b-κ and 3BC6-B12: IgG2b-κ) were produced, and the IgG isotypes were determined. Immunoblot analysis was performed to determine whether the five mAbs are responsive to rPvMSP1P-19, which showed an approximately 14 kDa specific band (Fig. 2b). The naïve antigen recognition and subcellular localization of mAbs were confirmed by mature schizont of *P. vivax*. All of mAbs were found to be specific to recognize the native parasite antigen, however, the strength of recognition was different (Fig. 2c). As a surface antigen control, the PvMSP1-19 polyclonal antibody was used to compare the co-localization. Unexpectedly, only 1BH9-A10 clearly overlapped with PvMSP1 (Fig. 2c). Another four mAbs, 2AF4-A2, 2AF4-A6, 3BC6-A5 and 3BC6-B12, showed diverse scattered recognition patterns on the merozoite surface (Fig. 2c). Different panels were showed with low magnification in *P. vivax* and *P. knowlesi* (Supplementary Figs S2 and S3).

### Linear epitopes of monoclonal antibodies

**Figure 3:**
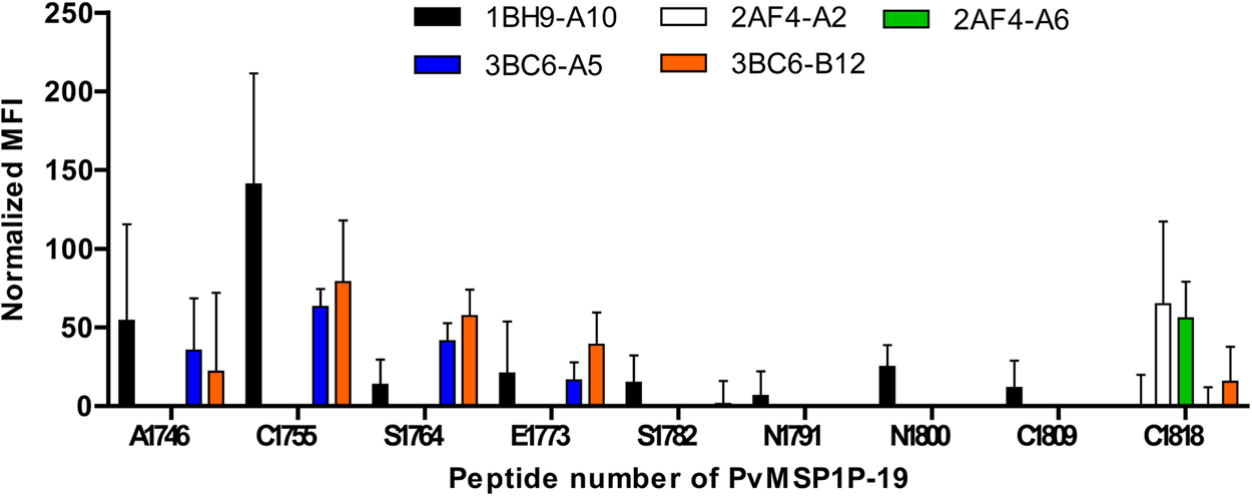
PvMSP1P-19 monoclonal antibody epitope mapping. The normalized mean fluorescence intensity (MFI) was calculated by comparing the no peptide printed slide well MFI value with each peptide printed array well MFI value. Data are shown as the mean ± S.D. of four independent experiments.

To investigate the linear epitopes of monoclonal antibodies, eighteen-mer overlapping peptides were synthesized (Fig. 1a) and spotted onto array slide. The spotted peptides were incubated with each monoclonal antibody at 1:200 and measured by goat anti-mouse Alexa Fluor 546 antibodies. 1BH9-A10, 3BC6-A5, and 3BC6-B12 were found to bind at the N-terminal region covering epitope peptides from A1746 to E1773 (Ala1746-Leu1790 in the amino acid position), and strongly recognized at C1755 (_1755_CRNRKCPLNSFCFIQTIN_1772_) (Fig. 3). In contrast, 2AF4-A2 and 2AF4-A6 recognized a single peptide C1818 (_1818_CICPKGTKPMHEGVVCSF_1835_) at the C-terminal region (Fig. 3).

### Erythrocyte binding inhibitory ability

**Figure 4:**
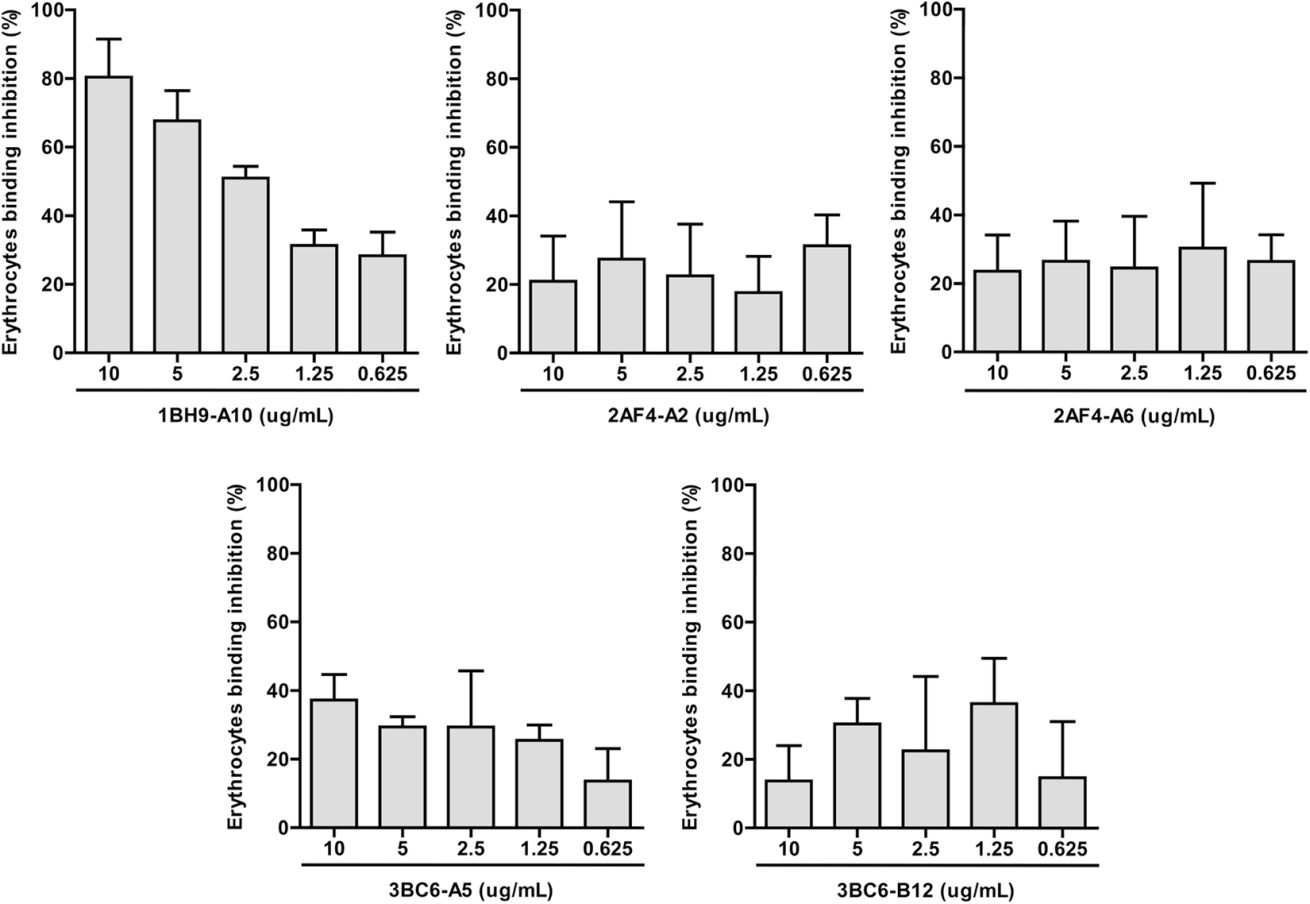
Erythrocyte binding inhibitory effect of PvMSP1P-19 monoclonal antibodies. The percentage of relative binding was calculated by comparing the number of rosettes between no antibody-treated and monoclonal antibody-treated samples. The PvMSP1P-19 specific monoclonal antibody, 1BH9-A10, showed red blood cell binding inhibitory activity. Data are shown as the mean ± S.D. of three independent experiments.

Erythrocyte binding inhibitory ability of PvMSP1P-19 specific antibody was confirmed previously^19,20^. The functional activity of five monoclonal antibodies were confirmed by erythrocyte binding inhibition in *in vitro* system. A serial dilution of each mAb and the non-treated control were used for erythrocyte binding inhibition activity confirmation. The inhibitory effects on mAbs were described as the percentage of erythrocyte binding inhibition compared to the mock control. 1BH9-A10 showed a binding inhibitory effect in a concentration-dependent manner (Fig. 4, Supplementary Fig. S4). However, 2AF4-A2, 2AF4-A6, 3BC6-A5, and 3BC6-B12 did not affect erythrocyte binding inhibition (Fig. 4).

### Cross-reactivity with *P. knowlesi*

**Figure 5:**
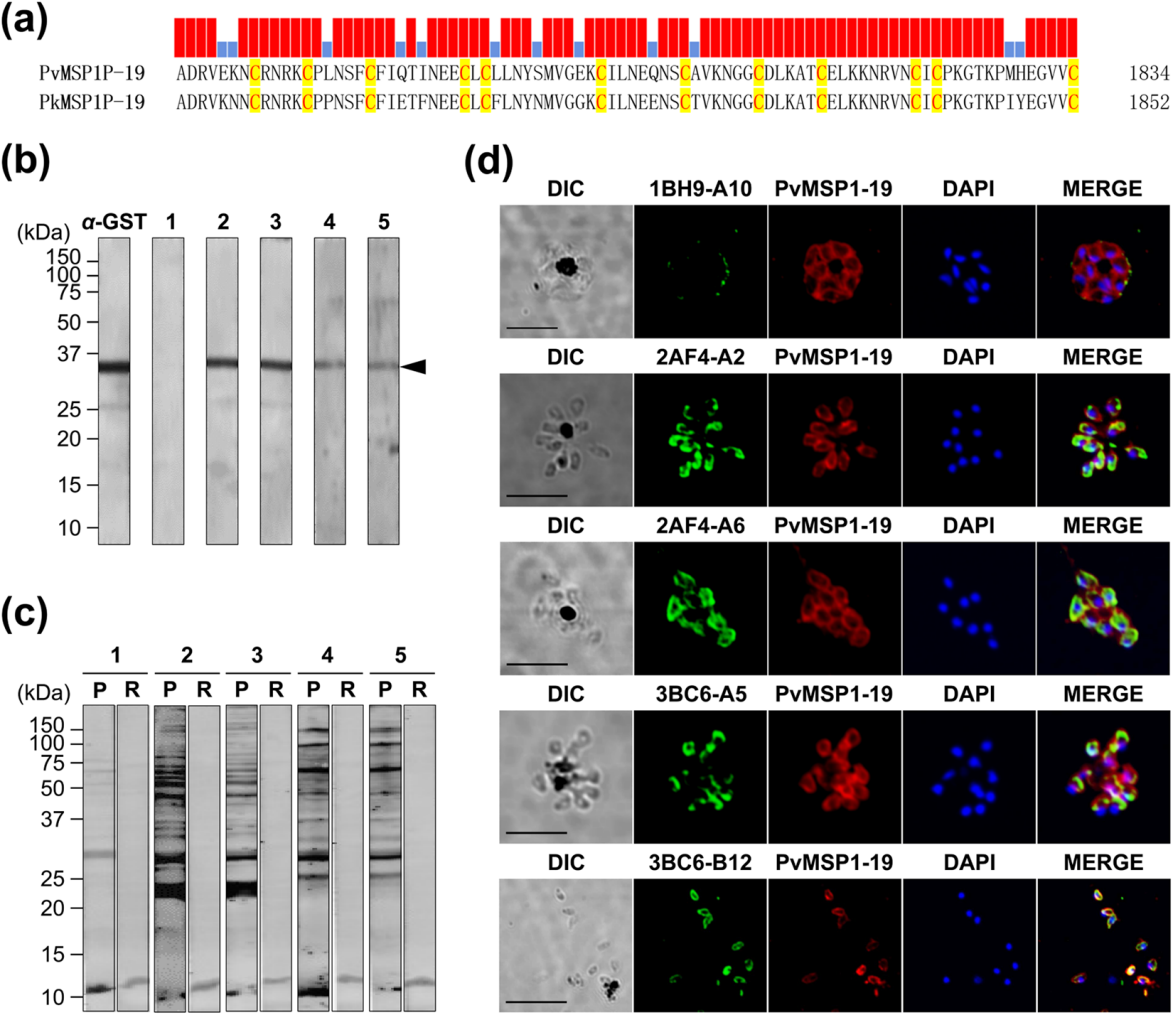
Cross-reactivity of PvMSP1P-19 monoclonal antibodies with *P. knowlesi*. (**a**) MSP1P EGF-like domain (MSP1P-19) amino-acid sequence comparison between PvMSP1 and PkMSP1. The red bar indicates identical sequence and blue bar indicates diverse sequence. (**b**) A western blot showing five monoclonal antibodies reacting with recombinant PkMSP1P-19. The approximately 37 kDa specific band indicates rPkMSP1P-19 (arrow head). GST, anti-GST antibody; lanes 1–5, anti-PvMSP1P-19 monoclonal antibodies as follows: lane 1, 1BH9-A10; lane 2, 2AF4-A2; lane 3, 2AF4-A6; lane 4, 3BC6-A5; and lane 5, 3BC6-B12. (**c**) A western blot showing five monoclonal antibodies reacting with *P. knowlesi* parasite lysate. The clear multiple band indicates processed naïve PkMSP1P-19. Lanes 1–5, anti-PvMSP1P-19 monoclonal antibodies as follows: lane 1, 1BH9-A10; lane 2, 2AF4-A2; lane 3, 2AF4-A6; lane 4, 3BC6-A5; and lane 5, 3BC6-B12. P, *P. knowlesi* lysate; R, normal RBC extract. (**d**) Reactivity observed by PvMSP1P-19 monoclonal antibodies with *P. knowlesi* merozoite at schizont stage by immunofluorescence assay. The mature schizont of *P. knowlesi* was dual labelled with PvMSP1P-19 monoclonal antibodies (green) and rabbit immune sera against PvMSP1-19 (red, merozoite surface marker). Nuclei are visualized with DAPI (blue). Bar indicate 5 μm.

*P. vivax* and *P. knowlesi* MSP1P EGF domains contained complete conservation of cysteine residues with high amino acids sequence similarity (86%)^24^ (Fig. 5a). Immunoblot analysis was performed with *P. knowlesi* derived recombinant MSP1P EGF domain and naïve parasite to determine cross-reactivity confirmation. Four mAbs clearly recognized the rPkMSP1P-19 and naïve *P. knowlesi* schizont lysate except 1BH9-A10 which has erythrocyte binding inhibition activity (Figs 5b,c). The naïve *P. knowlesi* subcellular recognition by the mAbs in the mature schizont were observed on the merozoite surface. However, 1BH9-A10 mAb showed poor recognition with *P. knowlesi* as it is consistent with immunoblotting (Fig. 5d, Supplementary Figs S2 and S3). It may suggest the species-specific recognition of 1BH9-A10 mAb to *P. vivax*.

### Parasite invasion inhibitory ability

To determine PvMSP1P-19 mAb functions to abrogate merozoite invasion, the human erythrocyte-adapted *P. knowlesi* parasite was used in *ex vivo* invasion inhibition assay. *P. knowlesi* parasites are used for vivax study model, especially during the blood stage, because of the high sequence similarity with conserved cysteine in the EGF-like domain and a feasible *in vitro* culture^24^.

**Figure 6:**
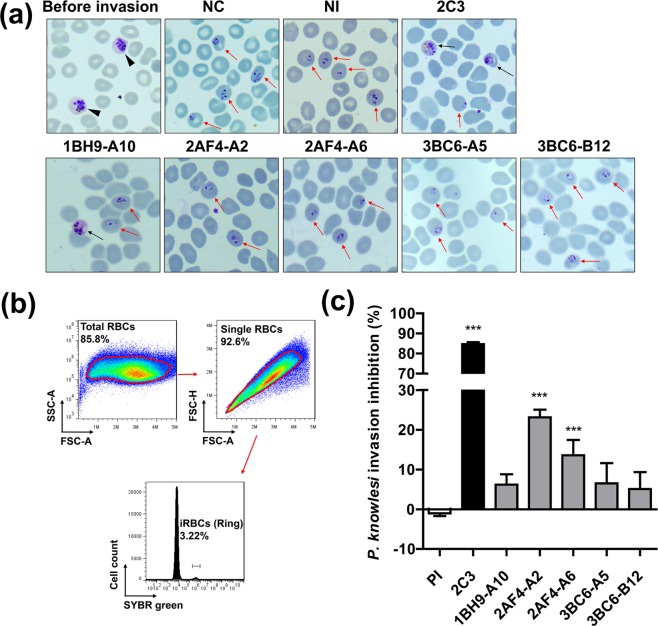
*P. knowlesi* invasion inhibition activities of PvMSP1P-19 monoclonal antibodies. (**a**) *P. knowlesi* stages were confirmed by morphology under light microscopy. The black arrow head indicates the mature schizont stage of *P. knowlesi* before invasion. The black arrow points to an unhealthy parasite, and the red arrow indicates the healthy ring stage of the parasite after re-invasion. (**b**) FACS gating strategy of ring stage parasitaemia evaluation. The schizont stage parasites are considered to have SYBR green signals of more than log 10^6^. (**c**) The *P. knowlesi* invasion inhibition efficacy was confirmed by an invasion inhibition assay. Data are shown as the invasion inhibition rate mean ± standard deviation (S.D.) with pre-immune sera (PI) and anti-2C3 (murine anti-Fy6) and PvMSP1P-19 monoclonal antibodies. Significant differences between PI and anti-2C3 or monoclonal antibodies calculated with a one-way ANOVA with the Tukey post-test. ***p < 0.001.

The parasites were cultured with or without mAb, and the healthy ring-form morphology of *P. knowlesi* was confirmed under microscopy (Fig. 6a). The healthy morphology of the parasites was reflected by the fact that the mAbs did not affect the parasite growth (Fig. 6a). FACS analysis was used for parasitaemia calculations with total cells (total RBCs) followed by single cells (single RBCs) using manual gating strategies. The single RBCs were considered feasible forms of *P. knowlesi* invasion, and the SYBR green range was set to less than log10^6^ to avoid the mature stage of parasites or monocyte contamination (Fig. 6b). Invasion inhibition activities of 2AF4-A2 (23.1 ± 2.0 in mean ± S.D.) and 2AF4-A2 (13.6 ± 3.8) were observed; however, 1BH9-A10 (6.2 ± 2.6), 3BC6-A5 (6.5 ± 5.1), and 3BC6-A5 (5.1 ± 4.2) did not inhibit parasite invasion significantly compared with pre-immune sera (PI, −1.0 ± 0.7) (Fig. 6c). Consistent with previous studies^25,26^, anti-DARC mAb (2C3, 84.7 ± 1.1) showed significant invasion inhibition activity.

### PvMSP1P-19 linear B-cell epitope in *P. vivax* patients

The B-cell linear epitopes of PvMSP1P in natural infection were identified by peptide array with vivax patient sera (Table 1) at 1:25 dilution and visualized by goat anti-human Alexa Fluor 546 antibodies. The normalization of the mean fluorescence intensity (MFI) of the average healthy group was compared to that of each peptide group (Table 2, Supplementary Fig. S5). Three peptides, S1764, E1773, and N1791, detected an epitope in the vivax patient that was in line with humoral immune reactivity (Table 2). The peptides C1809 (*p* = 0.0272) and C1818 (*p* = 0.0012) in the C-terminal region also showed significantly high IgG response in patient groups. Among them, E1773 showed the highest sero-positivity of 70% (1.80-fold higher than healthy individuals), followed by S1764 and N1791 at 53.3% (1.63-fold) and 50.0% (1.62-fold), respectively (Table 2). The C-terminal domain of peptides C1809 and C1818  showed only 20.0% (1.19-fold) and 26.7% (1.44-fold) sero-positivity. The first B-cell epitope in the vivax patient was determined to be at overlapping sites of S1764 and E1773, which are found in the short peptide _1773_EECLCLLNY_1781_. The second linear B-cell epitope was detected at _1791_NEQNSCAVKNGGCDLKAT_1808_. This linear epitope showed that only the complete sequence of N1791 can elicit an antibody response, which is at the central part of the sequence of the B-cell epitope.

**Table 2:** Sensitivity and specificity of PvMSP1P-19 B-cell epitopes in *P. vivax* patients and healthy individuals.

Peptide	No. of patient samples			95% CI^b^	MFI^c^	No. of healthy samples			95% CI	MFI	*p* value^e^	Fold increase
	+ve	− ve	Total (%)^a^			+ve	− ve	Total (%)^d^				
A1746	3	27	30 (10.0)	3.5–25.6	38744.2	2	28	30 (93.3)	78.7–98.2	34008.7	0.1060	1.14
C1755	2	28	30 (6.7)	1.9–21.3	24311.4	2	28	30 (93.3)	78.7–98.2	22898.6	0.4253	1.06
S1764	16	14	30 (53.3)	36.1–69.8	44943.8	1	29	30 (96.7)	83.3–99.4	27510.5	<*0.0001*	1.63
E1773	21	9	30 (70.0)	54.1–83.3	50022.2	1	29	30 (96.7)	83.3–99.4	27781.9	<*0.0001*	1.80
S1782	1	29	30 (3.3)	0.6–16.7	32749.3	0	30	30 (100.0)	88.7–100.0	30315.7	0.3558	1.08
N1791	15	15	30 (50.0)	33.2–66.9	35701.9	2	28	30 (93.3)	78.7–98.2	22036.6	<*0.0001*	1.62
N1800	3	27	30 (10.0)	3.5–25.6	30041.4	1	29	30 (96.7)	83.3–99.4	26401.4	0.0654	1.14
C1809	6	24	30 (20.0)	9.5–37.3	33644.1	0	30	30 (100.0)	88.7–100.0	28395.4	0.0272	1.19
C1818	8	22	30 (26.7)	14.2–44.5	15419.2	1	29	30 (96.7)	83.3–99.4	10728.0	0.0012	1.44

^a^Sensitivity: percentage of positive patient samples.

^b^CI: confidence interval.

^c^MFI: mean fluorescence intensity.

^d^Specificity: percentage of negative healthy samples.

^e^Differences in the total IgG prevalence for each antigen between vivax patients and healthy individuals were calculated using Student’s* t*-test. A *p* value < 0.05 is considered statistically significant.

## Discussion

According to the *P. vivax* research in previous reports, merozoite surface antigens such as PvMSP1, PvMSP3α, PvMSP3β, PvMSP8, PvMSP9, Pv92, and PvMSA180 elicited high antibodies responses^19,27–33^. The functional activity of antibodies in malaria has multiple roles, such as merozoite invasion inhibition, agglutination, growth inhibition, rosetting inhibition, opsonic phagocytosis, and direct killing by complement mediation^34^. Both *P. falciparum* and *P. vivax* express MSP1, a major antigen of the merozoite surface that is extensively studied. The antibody against PfMSP1 EGF-like domain (PfMSP1-19) was confirmed to inhibit invasion in previous reports^35,36^. Processing of MSP1 was also found to play role in parasite viability and merozoite egress^9^. Additionally, MSP1 has successfully mapped antibody epitopes and their roles in the two EGF-like domains^37^. In line with the MSP1 study, an antibody against the EGF-like domain of PvMSP1P shows functional activity for erythrocyte binding interruption^7,20,36,38^. Thus, as examined in this study, functional epitopes for immune responses and erythrocyte binding ability are important for understanding the biology of *P. vivax* in patients.

Five monoclonal antibodies were produced and confirmed using indirect fluorescence assay in naïve *P. vivax* and *P. knowlesi* parasites. The 2AF4-A2, 2AF4-A6, 3BC6-A5, and 3BC6-B12 staining pattern was scattered on the vivax merozoite surface. It might be due to that epitope does not expose or masking so that monoclonal antibodies could not recognize whole part of PvMSP1P-19 domain as polyclonal antibody does. Meanwhile, PvMSP1P-19 in the vivax patients was highly antigenic at the acute phase in 68.0% of ROK (*n* = 102) and 72.5% of Thailand (*n* = 40) isolates^19,39^. These antibodies were stable up to nine months after the patient recovered, and PvMSP1P-19 in the high responder was directly related to the erythrocyte binding inhibition in *in vitro* system, but not in low responder^39^. Direct erythrocyte binding inhibition ability showed that 1BH9-A10 mAb recognized the C1755 peptide. However, the C1755 peptide position failed to induce an immune response in most vivax patients. This result indicated that the high-responder patient IgG might contain the C1755 recognition antibodies. For parasite invasion inhibition, the effective B-cell epitope was identified in the C-terminal domain as _1818_CICPKGTKPMHEGVVCSF_1835_. In this position, IgG expression was induced in the patient; however, low sero-positivity (26.7%) was observed. Due to the difficulty to perform invasion inhibition assay with *P. vivax*, the invasion inhibition assay was performed with *P. knowlesi*. *P. knowlesi* and *P. vivax* showed high homology rate in various antigens including *msp1p* gene which can be used as an alternative way for vivax study model^24,40^. However, 1BH9-A10 which erythrocyte binding inhibitory mAb did not recognize in *P. knowlesi*. This result indicated that 1BH9-A10 recognition site was hampered by the polymorphic residues between orthologue at the N terminus. Additionally, the discrepancy of erythrocyte binding and invasion inhibition result might be caused by following reasons. The invasion inhibitory mAb was also observed in PfMSP1 by inhibition of processing of PfMSP1-42^36^. PvMSP1P also showed processing^19^, and the mAbs 2AF4-A2 and 2AF4-A6 could inhibit the processing of PvMSP1P. Additionally, previous studies indicate that direct binding and antibody recognition sites are not related^36,41^. The parasite growth inhibition ability of mAbs was confirmed by morphological examination. The morphology under the mAb treatment condition was maintained in the healthy condition, which indicates that mAbs against PvMSP1P-19 have no effect on parasite growth.

The merozoite surface antigen has been identified as a promising vaccine candidate from the malaria asexual stage^42^. Generally, merozoite surface antigens have a major role in invasion as a trigger for initial attachment to host target red blood cells^10,30,43^. Because of surface localization, genetic polymorphisms frequently occur by selection from host immune pressure in natural infection^44^. These polymorphisms are raising a problem in vaccine trials^45^. However, a surface antigen remains a promising target for a malaria vaccine because the merozoite surface antigens have a longer exposure time until internalization. To overcome the low efficacy of the malaria vaccine, multiple antigen combinations seem to be a good alternative^46–48^. Along with these combinations, the antigen that has resistance to host immune pressure in nature and elicits a high humoral immune response will be designed as a single or multiple vaccine candidate. PvMSP1P-19 confirmed that worldwide isolate sequences were found limited polymorphism. The conserved sequences will avoid vaccine efficiency problems from allele specificity. Taken together, PvMSP1P-19 elicits stable and high IgG responses in vivax patients regardless of polymorphisms from host immune pressure. The functions of antibodies showed possibility of parasite invasion abrogation. In summary, two effective epitopes were identified at _1755_CRNRKCPLNSFCFIQTIN_1772_ and _1818_CICPKGTKPMHEGVVCSF_1835_ for disrupting of PvMSP1P with erythrocytes interaction. However, these epitope regions should overcome the lack of natural boosting. Thus, we propose that PvMSP1P can be considered for a vaccine design strategy either for multiple or single vaccine development.

## Materials and Methods

### Sample collection and ethical clearance

The blood samples were collected in three malaria-endemic areas of the Republic of Korea (ROK, *n* = 11), Thailand (*n* = 7) and Myanmar (*n* = 12) from 2014 to 2016, 2013 and 2012, respectively (Table 1). The vivax patient samples were confirmed immediately at the study site by malaria antigen using the P.f/Pan rapid diagnosis test kit (SDFK60) (SD Diagnostics, Giheung, Korea) and Giemsa-stained thin smears under microscopy. The healthy individual sera were collected from children under 10 years old with no malaria history in local hospitals of non-endemic areas, ROK. All experiments were performed in accordance with relevant guidelines and regulations and all experimental protocols involving human samples approved by the ethics committees of the Kangwon National University Hospital in the ROK (KWNUIRB-2016-04-005), the Faculty of Tropical Medicine, Mahidol University in Thailand (MUIRB2012/079.2408), and the Department of Medical Research, Republic of the Union of Myanmar (Approval No-52/Ethics, 2012). This study was approved by the Institutional Review Board at Kangwon National University Hospital. Written informed consent was obtained from all subjects and their guardians.

### Genetic diversity analysis

Thirty isolates from ROK, Thailand and Myanmar were used to determine sequence diversity of *pvmsp1p* along with PlasmoDB database in this study. *P. vivax* genomic DNA was extracted from whole blood samples of vivax malaria patients using the QIAamp DNA Blood Mini Kit (QIAGEN, Hilden, Germany) according to the manufacturer’s protocol. The *pvmsp1p-19* gene was amplified by the forward primer (5′-GACACCCTACACACAATCAACACT-3′) and reverse primer (5′- CTACGCAGTGACGAACGCGAGG-3′) with AccuPower^®^ *Pfu* PCR premix (Bioneer, Seoul, ROK) polymerase. The amplicon nucleotide sequence was analysed by the internal primer (5′-TGAAGTGCAACACGTGGAAT-3′) using an ABI 3700 Genetic Analyzer (Genotech, Daejeon, ROK). All the raw sequences were analysed and trimmed using the SeqMan software, Lasergene ver. 7.0 (DNASTAR, Madison, WI, USA).

Sequence diversity (*π*) and graphical visualization of thirty isolates from newly sequenced in this study and sixty-six *pvmsp1p* worldwide sequence from PlasmoDB were analysed using the sliding window option with window length 100 and step size 25 sites in DNAsp ver. 5.0 software.

### Accession numbers

The nucleotide sequences of PvMSP1P-19 sequences are available under GenBank Accession Numbers MF968906 to MF968935.

### Monoclonal antibody production

Recombinant PvMSP1P-19 (rPvMSP1P-19) was expressed by the wheat germ cell-free system (WGCF) (Cell-free Science, Matsuyama, Japan) and recombinant protein was confirmed by 12% sodium dodecyl sulfate-polyacrylamide gel electrophoresis (SDS-PAGE) as described previously^19^. Briefly, pEU-E01-His-TEV-MCS (Cell-free Science) vector were cloned with PvMSP1P-19 fragment. The recombinant protein expression was scaled up as large scale WGCF expression system manufacture’s protocol and purified with Ni-affinity chromatography. For monoclonal antibody production, female BALB/c mice were immunized intravenously with an rPvMSP1P-19 mixture containing Freund’s complete adjuvant (Sigma-Aldrich, St. Louis, MO, USA) and boosted at a two-week interval with an rPvMSP1P-19 mixture containing Freund’s incomplete adjuvant (Sigma-Aldrich). Four days later, anti-PvMSP1P-19 polyclonal antibodies were collected, and the spleen was used to produce hybridoma cell lines, as done previously^49,50^. Hybridoma culture supernatants were screened for antibody reactivity by enzyme-linked immunosorbent assay (ELISA) and indirect immunofluorescence assay (IFA). Positive cells were cloned by two rounds of limiting dilution, and the antibody isotype was determined using a monoclonal antibody isotyping kit (Santa Cruz Biotechnology, Inc., Santa Cruz, CA, USA) according to the manufacturer’s protocol. The cloned cell lines were expanded as ascites in mice primed with pristane (Wako Pure Chemical Industries, Osaka, Japan). Immunoglobulin G (IgG) was purified from the ascitic fluid using the MAbTrap Kit by following the manufacturer’s protocol (GE Healthcare, Little Chalfont, UK).

### Erythrocyte binding inhibition assay

The PvMSP1P-19 construct and associated primers were described previously^19^. Briefly, the PvMSP1P-19-containing pEGFP-HSVgD vector was used for target expression on the COS-7 cell surface. The PvMSP1P-19 amplicon was ligated using the In-Fusion^®^ HD Cloning Kit (Clontech, Palo Alto, CA, USA). This construct was cloned in JM109 competent cells (Real Biotech Corporation, Taiwan), and plasmid DNA was purified by the Ultrapure plasmid extraction system (Viogene, Taipei, Taiwan) according to the manufacturer’s protocol.

COS-7 cells were cultured in 24-well culture plates (Corning Inc., NY, USA) and transfected with 100 ng of PvMSP1P-19 construct in each well by Lipofectamine^®^ 2000 (Invitrogen, Carlsbad, CA, USA). At 42 hours post-transfection, COS-7 cells were pre-incubated with 10 ug/ml to 0.625 ug/ml dilutions of monoclonal antibodies for 1 hour at 37 °C. The monoclonal antibodies were washed out with PBS, and COS-7 cells were incubated with 10% haematocrit (*Htc*.) of erythrocytes in incomplete DMEM for 2 hours at 37 °C. Unbound erythrocytes were washed five times with PBS, and the number of rosettes in 30 fields was counted under microscopy using a 200x objectives lens. The erythrocyte binding inhibition abilities of the monoclonal antibody was compared with the un-treated control. A positive rosette was counted when adherent erythrocytes covered more than 50% of the COS-7 cell surface. The protein expression level was determined by detecting green fluorescence protein (GFP) in unfixed cells on a Fluoview^®^ FV1000 Laser Scanning Confocal Imaging System (Olympus, Tokyo, Japan) under the 20x objective lens.

### Peptide synthesis

The *P. vivax* Salvador-I strain sequence was used for synthesis of PvMSP1P-19 peptides. Sequential 18-mer peptides on 9-mer overlapping amino acids were chemically synthesized using Multiple Peptide Synthesis techniques in Solid Phase. The identity and purity of the peptides were analysed by analytical reversed phase-high-performance liquid chromatography (RP-HPLC) and mass spectrometry MALDI-TOF. All peptides solubilized in DMSO with more than 90% purity. The peptide sequence and information are described in Fig. 1a.

### Mapping the linear epitope for monoclonal antibodies and clinical isolates

Three-aminopropyl-coated glass slides were prepared as described previously^4^. Each peptide was labelled with Cy5 NHS-Ester (GE Healthcare) for array slide printing efficiency calculation. The peptides were diluted with 100 mM sodium bicarbonate buffer (pH 8.3) and 2 µg of Cy5 NHS-Ester (GE Healthcare) and incubated on ice for 2 hours. The reaction mixture was quenched with 1 M Tris-HCl (pH 8.0) solution and purified by Sephadex G-25 columns (GE Healthcare). The labelled peptides were diluted with 400 mM 1-ethyl-3-(3-dimethylaminopropyl) carbodiimide (EDC; Thermo Fisher Scientific Inc., Rochester, NY, USA) and 100 mM N-hydroxysuccinimide (NHS; Thermo Fisher Scientific Inc.) for peptide coupling to the amine slide. A total of 100 ng/μl of each peptide mixture was printed on the amine slide spot and incubated for 30 minutes at room temperature. The peptide printing efficiency was calculated by the Odyssey infrared imaging system (LI-COR Bioscience). The peptide printed array slides were probed with 1:200 dilution monoclonal antibodies for determining the monoclonal antibody binding epitope or probed with a 1:25 dilution of patient sera to identify the PvMSP1P-19 B-cell epitope in vivax patient. The arrays for visualization were incubated with 50 ng/μl goat anti-mouse and -human Alexa Fluor 546 antibodies (Invitrogen) and scanned in an Innoscan-300 (Innopsys, Carbonne, France).

### *P. knowlesi in vitro* culture and invasion inhibition assay

The human erythrocyte-adapted *P. knowlesi* A1-H.1 strain (PkA1-H.1, a kind gift from Robert W. Moon, LSHTM)^51^ was cultured with erythrocytes from O^+^ type blood samples. The parasite was cultured in RPMI-1640 medium containing sodium bicarbonate, dextrose anhydrous, hypoxanthine, Albumax II (0.05%), pooled human AB sera (10%) and Gentamicin. The schizont stage parasite was synchronized by magnetic-activated cell sorting (MACS) (Miltenyi Biotec, Bergisch Gladbach, Germany) with an LD column (Miltenyi Biotec) and sub-cultured for 10 hours with fresh medium, erythrocytes and 100 μg/ml PvMSP1P-19 monoclonal antibodies. The sub-cultured parasites were adjusted to 1.5% parasitaemia with 2% haematocrit. Additionally, 25 μg/mL DARC monoclonal antibody (2C3) (a kind gift from Renia L, Singapore Immunology Network-BMSI-A STAR) was used as an invasion blocking control^25,26^. After invasion, the ring stage was examined by thin smear with Giemsa staining under light microscopy and fluorescence-activated cell sorting (FACS). For FACS analysis, newly infected RBCs were fixed with glutaraldehyde (0.05%) and stained with SYBR green (Invitrogen). The data were analysed by a FACS Accuri™ C6 Flow Cytometer (Becton-Dickinson Co., Mansfield, MA, USA) with 200,000 total cell events.

### Immunoblot analysis

The PvMSP1P-19 recombinant protein (rPvMSP1P-19) with His-tag^19^, rPkMSP1P-19 with GST-tag (unpublished data) and *P. knowlesi* schizont lysate were separated by 13% SDS-PAGE and transferred to a PVDF membrane (Millipore, Bedford, MA, USA) by electrophoresis. The primary antibody was used at 1:1,000 dilution of anti-Penta-His mouse antibody (QIAGEN, Hilden, Germany) for rPvMSP1P-19, 1:10,000 dilution of anti-GST mouse monoclonal antibody (Novagen, Madison, WI, USA) for rPkMSP1P-10 in PBS-T for 1 hour at 25 °C. After primary antibody reactions, the membrane was incubated with a 1:10,000 dilution of goat anti-mouse IRDye^®^800 Secondary Antibody (LI-COR Bioscience, Lincoln, NE, USA) in PBS-T for 1 hour at 25 °C. Data were measured using the Odyssey infrared imaging system (LI-COR Bioscience) and analysed with Odyssey software (LI-COR Bioscience).

### Immunofluorescence assay

*P. vivax* thin smears from Korean isolates schizonts and *P. knowlesi* A1-H.1 schizonts were prepared with ice cold acetone fixation, and the smears were blocked with 5% BSA in PBS for 30 minutes at 37 °C. The parasite smears were incubated with a 1:100 dilution of mice monoclonal antibodies and anti-PvMSP1-19 antibody from rabbit for 1 hour at 37 °C, followed by incubation with a 1:500 dilution of Alexa Fluor 488 conjugated goat anti-mouse IgG (Invitrogen) and Alexa Fluor 546 conjugated goat anti-rabbit IgG (Invitrogen) as secondary antibodies for 30 minutes at 37 °C. The parasite nuclei were stained with 2 μg/ml DAPI (4′,6-diamidino-2-phenylindole) in the secondary antibody mixture. Slides were mounted by ProLong^®^ Gold Antifade reagent (Invitrogen) and visualized by a Fluoview^®^ FV1000 Laser Scanning Confocal Imaging System (Olympus, Tokyo, Japan) under the 60x objective oil-immersion lens. Images were visualized by FV10-ASW 3.0 viewer software.

### Statistical analysis

The binding inhibition assay and peptide array data were analysed using GraphPad Prism (GraphPad Software, San Diego, CA, USA) and Microsoft Excel 2013 (Microsoft, Redmond, WA, USA). Student’s *t*-test was used to compare the experimentally measured values for each group of the erythrocyte binding inhibition on monoclonal antibodies and peptide array for the linear B-cell epitope in patients. The significance of the *P. knowlesi* invasion inhibition assay results was determined with one-way ANOVA with the Tukey post-test, and *p* < 0.05 was considered significant.

## Supplementary information

Supplementary information
