# Supplementary material for: Inhibition of parasite invasion by monoclonal antibody against epidermal growth factor-like domain of Plasmodium vivax merozoite surface protein 1 paralog
Source: Sci Rep. 2019 Mar 7;9:3906. doi: 10.1038/s41598-019-40321-2 (PMC6405985; doi:10.1038/s41598-019-40321-2)
Supplement: Supplementary file 1 — Supplementary information [file 41598_2019_40321_MOESM1_ESM.pdf]

Supplementary Information

Inhibition of parasite invasion by monoclonal antibody against epidermal growth factor-like domain of *Plasmodium vivax* merozoite surface protein 1 paralog

Jin-Hee Han[1,2], Yang Cheng[1], Fauzi Muh[1], Md Atique Ahmed[1], Jee-Sun Cho[3], Myat Htut Nyunt[4], Hye-Yoon Jeon[5], Kwon-Soo Ha[5], Sunghun Na[6], Won Sun Park[7], Seok-Ho Hong[8], Ho-Joon Shin[9], Bruce M. Russell[2,3], Eun-Taek Han[1*]

[1] Department of Medical Environmental Biology and Tropical Medicine, School of Medicine, Kangwon National University, Chuncheon, Gangwon-do, Republic of Korea

[2] Department of Microbiology and Immunology, University of Otago, Dunedin 9054, New Zealand

[3] Department of Microbiology, Yong Loo Lin School of Medicine, National University of Singapore, National University Health System, Singapore 117597, Singapore; Singapore Immunology Network (SIgN), A*STAR, Singapore138648, Singapore

[4] Department of Medical Research, Yangon, Myanmar

[5] Department of Cellular and Molecular Biology, School of Medicine, Kangwon National University, Chuncheon, Gangwon-do, Republic of Korea

[6] Department of Obstetrics and Gynecology, School of Medicine, Kangwon National University, Chuncheon, Gangwon-do, Republic of Korea

[7] Department of Physiology, School of Medicine, Kangwon National University, Chuncheon, Gangwon-do, Republic of Korea

[8] Department of Internal Medicine, School of Medicine, Kangwon national University, Chuncheon, Gangwon-do, Republic of Korea

[9] Department of Microbiology, Ajou University School of Medicine, and Department of Biomedical Science, Ajou University Graduate School of Medicine, Suwon, Gyeonggi-do, Republic of Korea

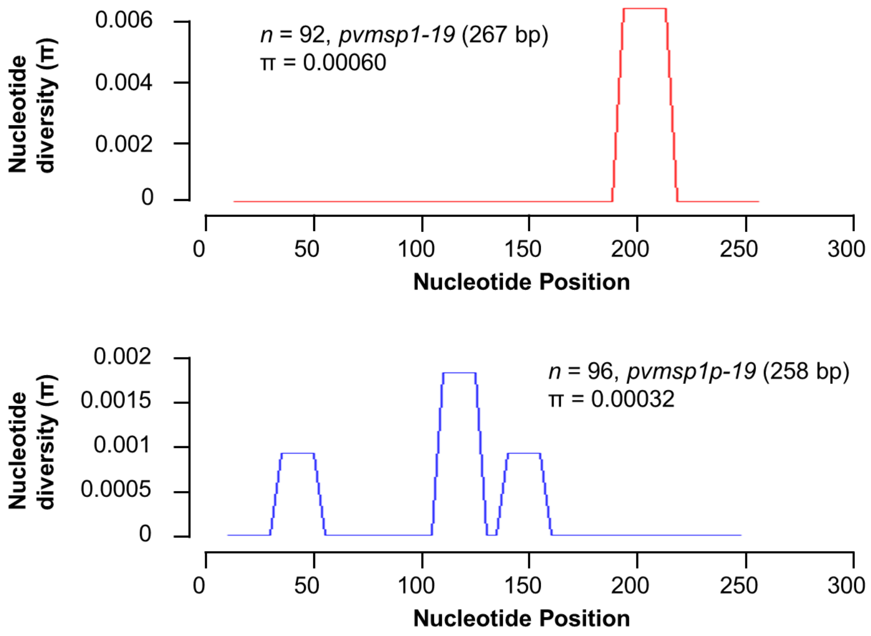

Supplementary Figure S1. The *pvmsp1-19* and *pvmsp1p-19* nucleotide diversity. *pvmsp1* (*n*=92) and *pvmsp1p* (*n*=66) isolates were obtained from PlasmoDB originating from 10 countries (Brazil, China, Columbia, India, Mauritania, Mexico, North Korea, Peru, Papua New Guinea, and Thailand) and *pvmsp1p-19* (*n*=30) sequence from ROK, Thailand and Myanmar were used for nucleotide diversity analysis. The nucleotide diversity (*π*) of *pvmsp1-19* was 0.00060 and *pvmsp1p-19* was 0.00032, thus indicating that *pvmsp1p*-19 was limited polymorphism worldwide. Graphical visualization was analyzed using the sliding window option with window length 20 and step size 5 site in DNAsp ver. 5.0 software.

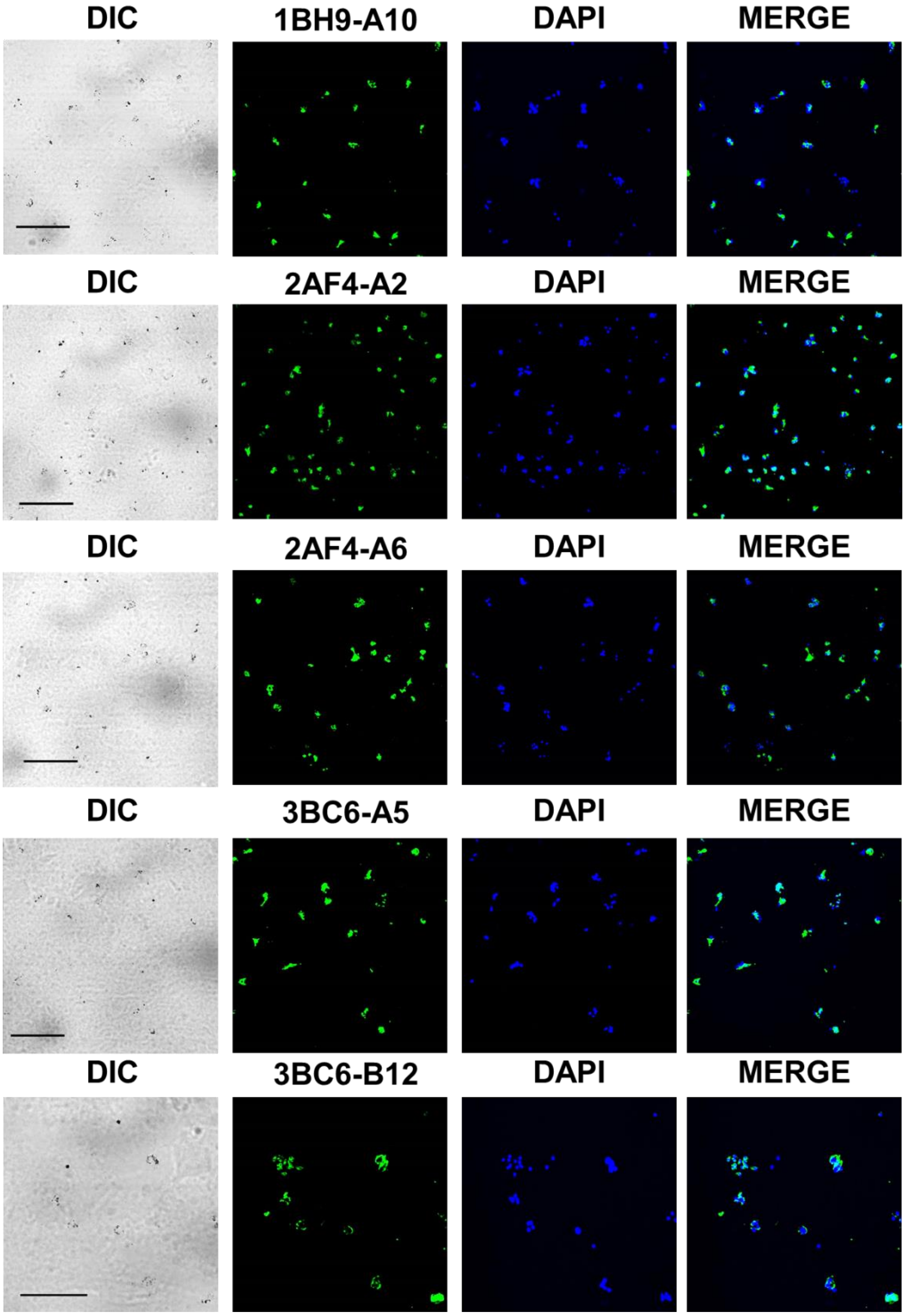

Supplementary Figure S2. The monoclonal antibodies recognition of *P. knowlesi* parasite on immunofluorescence assay. The mature schizont of *P. knowlesi* was labelled with PvMSP1P-19 monoclonal antibodies (green). Nuclei are visualized with DAPI (blue). Bars indicate 25 μm.

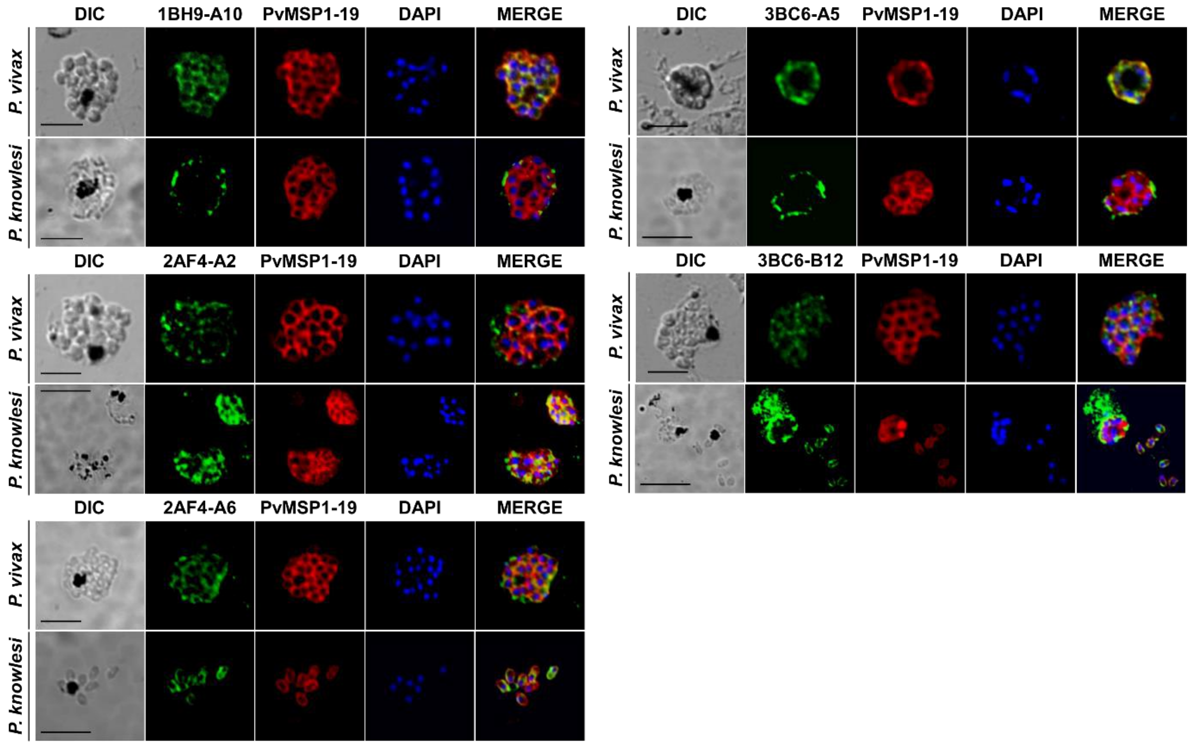

Supplementary Figure S3. *P. vivax* and *P. knowlesi* Immunofluorescence assay. The recognition of monoclonal antibodies with naïve PvMSP1P and PkMSP1P by immunofluorescence assay. The mature schizont of *P. vivax* and *P. knowlesi* was dual labelled with PvMSP1P-19 monoclonal antibodies (green) and rabbit immune sera against PvMSP1-19 (red, merozoite surface marker). Nuclei are visualized with DAPI (blue). Bars indicate 5 μm.

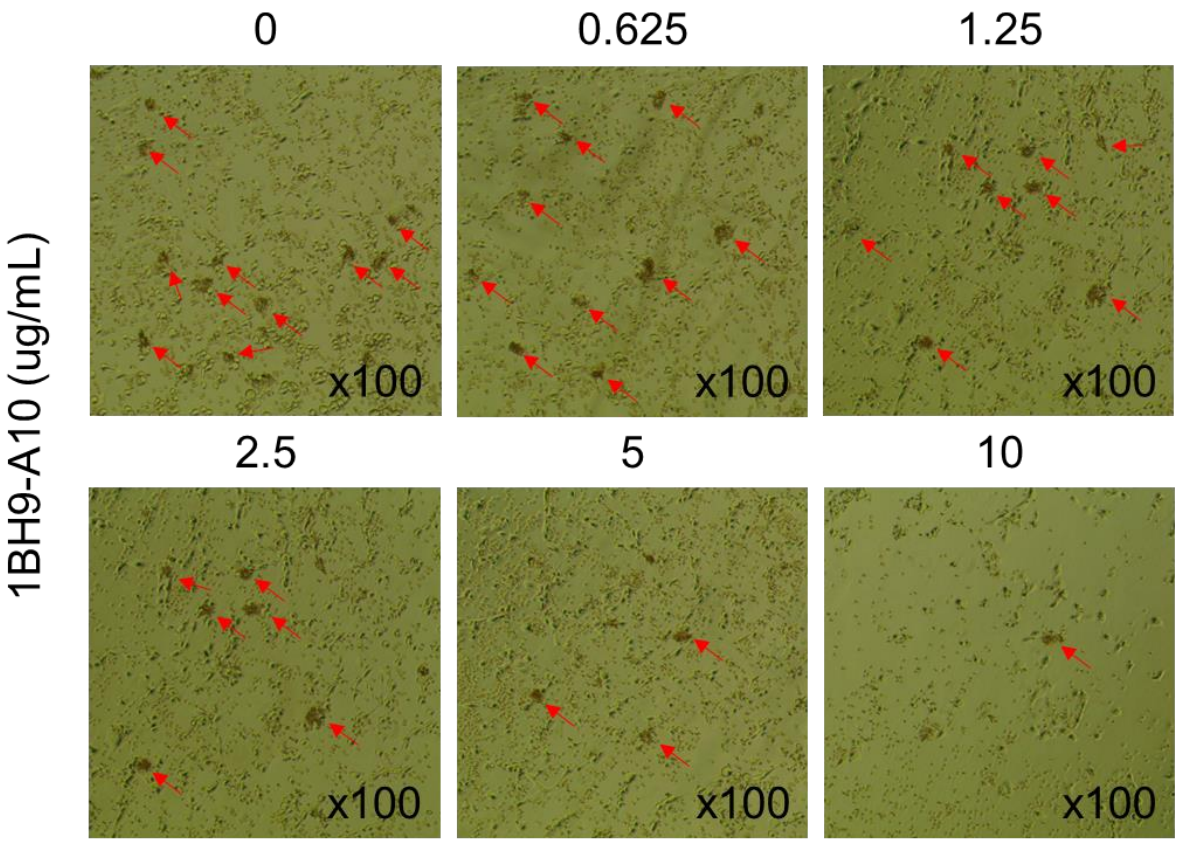

Supplementary Figure S4. The rosette formation of PvMSP1P-19. Erythrocyte binding rosettes formed on the surfaces of COS-7 cells expressing PvMSP1P-19 under the serial diluted 1BH9-A10. The red arrow indicates positive rosette formation.

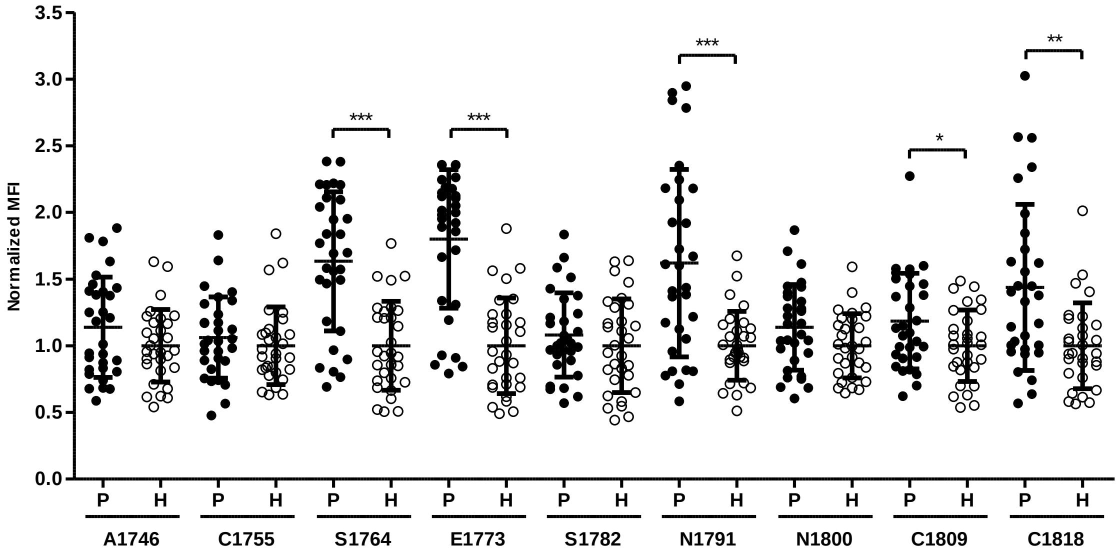

Supplementary Figure S5. B-cell epitope of PvMSP1P-19 on vivax patient. Thirty vivax malaria patient serum and healthy individual serum was used for PvMSP1P-19 B-cell epitope screening. The normalized MFI was calculated by each peptide with the patient MFI divided by the average value of healthy individual MFI for comparison of each peptide reactivity. Significant differences are shown as single asterisks, $p < 0.05$, double asterisks, $p < 0.01$ and triple asterisks, $p < 0.0001$ by student *t*-test. P, vivax-infected patient serum; H, healthy individual serum.

Supplement Dataset 1. Thirty of *pvmsp1p-19*sequence from Republic of Korea (KOR), Myanmar (MYA) and Thailand (THA). *Pvmsp1p-19*nucleotide sequences are available under GenBank accession numbers MF968906 to MF968935.

```
                            ---------+---------+---------+---------+---------+---------+---------+---------+
                                    10        20        30        40        50        60        70        80
                            ---------+---------+---------+---------+---------+---------+---------+---------+
(PVX_099975) PvMSP1P-19     GACCGTGTGGAAAAGAACTGCAGAAATAGGAAGTGCCCACTCAACTCCTTCTGCTTCATTCAAACGATTAATGAAGAGTG
(KOR) 201401_PvMSP1P-19     GACCGTGTGGAAAAGAACTGCAGAAATAGGAAGTGCCCACTCAACTCCTTCTGCTTCATTCAAACGATTAATGAAGAGTG
(KOR) 201402_PvMSP1P-19     GACCGTGTGGAAAAGAACTGCAGAAATAGGAAGTGCCCACTCAACTCCTTCTGCTTCATTCAAACGATTAATGAAGAGTG
(KOR) 201404_PvMSP1P-19     GACCGTGTGGAAAAGAACTGCAGAAATAGGAAGTGCCCACTCAACTCCTTCTGCTTCATTCAAACGATTAATGAAGAGTG
(KOR) 201502_PvMSP1P-19     GACCGTGTGGAAAAGAACTGCAGAAATAGGAAGTGCCCACTCAACTCCTTCTGCTTCATTCAAACGATTAATGAAGAGTG
(KOR) 201503_PvMSP1P-19     GACCGTGTGGAAAAGAACTGCAGAAATAGGAAGTGCCCACTCAACTCCTTCTGCTTCATTCAAACGATTAATGAAGAGTG
(KOR) 201504_PvMSP1P-19     GACCGTGTGGAAAAGAACTGCAGAAATAGGAAGTGCCCACTCAACTCCTTCTGCTTCATTCAAACGATTAATGAAGAGTG
(KOR) 201505_PvMSP1P-19     GACCGTGTGGAAAAGAACTGCAGAAATAGGAAGTGCCCACTCAACTCCTTCTGCTTCATTCAAACGATTAATGAAGAGTG
(KOR) 201602_PvMSP1P-19     GACCGTGTGGAAAAGAACTGCAGAAATAGGAAGTGCCCACTCAACTCCTTCTGCTTCATTCAAACGATTAATGAAGAGTG
(KOR) 201603_PvMSP1P-19     GACCGTGTGGAAAAGAACTGCAGAAATAGGAAGTGCCCACTCAACTCCTTCTGCTTCATTCAAACGATTAATGAAGAGTG
(KOR) 201604_PvMSP1P-19     GACCGTGTGGAAAAGAACTGCAGAAATAGGAAGTGCCCACTCAACTCCTTCTGCTTCATTCAAACGATTAATGAAGAGTG
(KOR) 201605_PvMSP1P-19     GACCGTGTGGAAAAGAACTGCAGAAATAGGAAGTGCCCACTCAACTCCTTCTGCTTCATTCAAACGATTAATGAAGAGTG
(MYA) KV01_PvMSP1P-19       GACCGTGTGGAAAAGAACTGCAGAAATAGGAAGTGCCCACTCAACTCCTTCTGCTTCATTCAAACGATTAATGAAGAGTG
(MYA) KV02_PvMSP1P-19       GACCGTGTGGAAAAGAACTGCAGAAATAGGAAGTGCCCACTCAACTCCTTCTGCTTCATTCAAACGATTAATGAAGAGTG
(MYA) KV03_PvMSP1P-19       GACCGTGTGGAAAAGAACTGCAGAAATAGGAAGTGCCCACTCAACTCCTTCTGCTTCATTCAAACGATTAATGAAGAGTG
(MYA) KV04_PvMSP1P-19       GACCGTGTGGAAAAGAACTGCAGAAATAGGAAGTGCCCACTCAACTCCTTCTGCTTCATTCAAACGATTAATGAAGAGTG
(MYA) KV07_PvMSP1P-19       GACCGTGTGGAAAAGAACTGCAGAAATAGGAAGTGCCCACTCAACTCCTTCTGCTTCATTCAAACGATTAATGAAGAGTG
(MYA) KV08_PvMSP1P-19       GACCGTGTGGAAAAGAACTGCAGAAATAGGAAGTGCCCACTCAACTCCTTCTGCTTCATTCAAACGATTAATGAAGAGTG
(MYA) KV09_PvMSP1P-19       GACCGTGTGGAAAAGAACTGCAGAAATAGGAAGTGCCCACTCAACTCCTTCTGCTTCATTCAAACGATTAATGAAGAGTG
(MYA) KV10_PvMSP1P-19       GACCGTGTGGAAAAGAACTGCAGAAATAGGAAGTGCCCACTCAACTCCTTCTGCTTCATTCAAACGATTAATGAAGAGTG
(MYA) KV13_PvMSP1P-19       GACCGTGTGGAAAAGAACTGCAGAAATAGGAAGTGCCCACTCAACTCCTTCTGCTTCATTCAAACGATTAATGAAGAGTG
(MYA) KV14_PvMSP1P-19       GACCGTGTGGAAAAGAACTGCAGAAATAGGAAGTGCCCACTCAACTCCTTCTGCTTCATTCAAACGATTAATGAAGAGTG
(MYA) KV15_PvMSP1P-19       GACCGTGTGGAAAAGAACTGCAGAAATAGGAAGTGCCCACTCAACTCCTTCTGCTTCATTCAAACGATTAATGAAGAGTG
(MYA) KV16_PvMSP1P-19       GACCGTGTGGAAAAGAACTGCAGAAATAGGAAGTGCCCACTCAACTCCTTCTGCTTCATTCAAACGATTAATGAAGAGTG
(THA) LS02_PvMSP1P-19       GACCGTGTGGAAAAGAACTGCAGAAATAGGAAGTGCCCACTCAACTCCTTCTGCTTCATTCAAACGATTAATGAAGAGTG
(THA) PR01_PvMSP1P-19       GACCGTGTGGAAAAGAACTGCAGAAATAGGAAGTGCCCACTCAACTCCTTCTGCTTCATTCAAACGATTAATGAAGAGTG
(THA) TS02_PvMSP1P-19       GACCGTGTGGAAAAGAACTGCAGAAATAGGAAGTGCCCACTCAACTCCTTCTGCTTCATTCAAACGATTAATGAAGAGTG
(THA) TS03_PvMSP1P-19       GACCGTGTGGAAAAGAACTGCAGAAATAGGAAGTGCCCACTCAACTCCTTCTGCTTCATTCAAACGATTAATGAAGAGTG
(THA) TS04_PvMSP1P-19       GACCGTGTGGAAAAGAACTGCAGAAATAGGAAGTGCCCACTCAACTCCTTCTGCTTCATTCAAACGATTAATGAAGAGTG
(THA) TS05_PvMSP1P-19       GACCGTGTGGAAAAGAACTGCAGAAATAGGAAGTGCCCACTCAACTCCTTCTGCTTCATTCAAACGATTAATGAAGAGTG
(THA) TS06_PvMSP1P-19       GACCGTGTGGAAAAGAACTGCAGAAATAGGAAGTGCCCACTCAACTCCTTCTGCTTCATTCAAACGATTAATGAAGAGTG
```

```
                             ---------+---------+---------+---------+---------+---------+---------+---------+
                                     90       100       110       120       130       140       150       160
                             ---------+---------+---------+---------+---------+---------+---------+---------+
(PVX_099975) PvMSP1P-19      CCTGTGCCTCCTTAACTACAGCATGGTGGGGGAGAAGTGCATTTTAAACGAACAGAATTCTTGCGCGGTGAAAAACGGGG
(KOR) 201401_PvMSP1P-19      CCTGTGCCTCCTTAACTACAGCATGGTGGGGGAGAAGTGCATTTTAAACGAACAGAATTCTTGCGCGGTGAAAAACGGGG
(KOR) 201402_PvMSP1P-19      CCTGTGCCTCCTTAACTACAGCATGGTGGGGGAGAAGTGCATTTTAAACGAACAGAATTCTTGCGCGGTGAAAAACGGGG
(KOR) 201404_PvMSP1P-19      CCTGTGCCTCCTTAACTACAGCATGGTGGGGGAGAAGTGCATTTTAAACGAACAGAATTCTTGCGCGGTGAAAAACGGGG
(KOR) 201502_PvMSP1P-19      CCTGTGCCTCCTTAACTACAGCATGGTGGGGGAGAAGTGCATTTTAAACGAACAGAATTCTTGCGCGGTGAAAAACGGGG
(KOR) 201503_PvMSP1P-19      CCTGTGCCTCCTTAACTACAGCATGGTGGGGGAGAAGTGCATTTTAAACGAACAGAATTCTTGCGCGGTGAAAAACGGGG
(KOR) 201504_PvMSP1P-19      CCTGTGCCTCCTTAACTACAGCATGGTGGGGGAGAAGTGCATTTTAAACGAACAGAATTCTTGCGCGGTGAAAAACGGGG
(KOR) 201505_PvMSP1P-19      CCTGTGCCTCCTTAACTACAGCATGGTGGGGGAGAAGTGCATTTTAAACGAACAGAATTCTTGCGCGGTGAAAAACGGGG
(KOR) 201602_PvMSP1P-19      CCTGTGCCTCCTTAACTACAGCATGGTGGGGGAGAAGTGCATTTTAAACGAACAGAATTCTTGCGCGGTGAAAAACGGGG
(KOR) 201603_PvMSP1P-19      CCTGTGCCTCCTTAACTACAGCATGGTGGGGGAGAAGTGCATTTTAAACGAACAGAATTCTTGCGCGGTGAAAAACGGGG
(KOR) 201604_PvMSP1P-19      CCTGTGCCTCCTTAACTACAGCATGGTGGGGGAGAAGTGCATTTTAAACGAACAGAATTCTTGCGCGGTGAAAAACGGGG
(KOR) 201605_PvMSP1P-19      CCTGTGCCTCCTTAACTACAGCATGGTGGGGGAGAAGTGCATTTTAAACGAACAGAATTCTTGCGCGGTGAAAAACGGGG
(MYA) KV01_PvMSP1P-19        CCTGTGCCTCCTTAACTACAGCATGGTGGGGGAGAAGTGCATTTTAAACGAACAGAATTCTTGCGCGGTGAAAAACGGGG
(MYA) KV02_PvMSP1P-19        CCTGTGCCTCCTTAACTACAGCATGGTGGGGGAGAAGTGCATTTTAAACGAACAGAATTCTTGCGCGGTGAAAAACGGGG
(MYA) KV03_PvMSP1P-19        CCTGTGCCTCCTTAACTACAGCATGGTGGGGGAGAAGTGCATTTTAAACGAACAGAATTCTTGCGCGGTGAAAAACGGGG
(MYA) KV04_PvMSP1P-19        CCTGTGCCTCCTTAACTACAGCATGGTGGGGGAGAAGTGCATTTTAAACGAACAGAATTCTTGCGCGGTGAAAAACGGGG
(MYA) KV07_PvMSP1P-19        CCTGTGCCTCCTTAACTACAGCATGGTGGGGGAGAAGTGCATTTTAAACGAACAGAATTCTTGCGCGGTGAAAAACGGGG
(MYA) KV08_PvMSP1P-19        CCTGTGCCTCCTTAACTACAGCATGGTGGGGGAGAAGTGCATTTTAAACGAACAGAATTCTTGCGCGGTGAAAAACGGGG
(MYA) KV09_PvMSP1P-19        CCTGTGCCTCCTTAACTACAGCATGGTGGGGGAGAAGTGCATTTTAAACGAACAGAATTCTTGCGCGGTGAAAAACGGGG
(MYA) KV10_PvMSP1P-19        CCTGTGCCTCCTTAACTACAGCATGGTGGGGGAGAAGTGCATTTTAAACGAACAGAATTCTTGCGCGGTGAAAAACGGGG
(MYA) KV13_PvMSP1P-19        CCTGTGCCTCCTTAACTACAGCATGGTGGGGGAGAAGTGCATTTTAAACGAACAGAATTCTTGCGCGGTGAAAAACGGGG
(MYA) KV14_PvMSP1P-19        CCTGTGCCTCCTTAACTACAGCATGGTGGGGGAGAAGTGCATTTTAAACGAACAGAATTCTTGCGCGGTGAAAAACGGGG
(MYA) KV15_PvMSP1P-19        CCTGTGCCTCCTTAACTACAGCATGGTGGGGGAGAAGTGCATTTTAAACGAACAGAATTCTTGCGCGGTGAAAAACGGGG
(MYA) KV16_PvMSP1P-19        CCTGTGCCTCCTTAACTACAGCATGGTGGGGGAGAAGTGCATTTTAAACGAACAGAATTCTTGCGCGGTGAAAAACGGGG
(THA) LS02_PvMSP1P-19        CCTGTGCCTCCTTAACTACAGCATGGTGGGGGAGAAGTGCATTTTAAACGAACAGAATTCTTGCGCGGTGAAAAACGGGG
(THA) PR01_PvMSP1P-19        CCTGTGCCTCCTTAACTACAGCATGGTGGGGGAGAAGTGCATTTTAAACGAACAGAATTCTTGCGCGGTGAAAAACGGGG
(THA) TS02_PvMSP1P-19        CCTGTGCCTCCTTAACTACAGCATGGTGGGGGAGAAGTGCATTTTAAACGAACAGAATTCTTGCGCGGTGAAAAACGGGG
(THA) TS03_PvMSP1P-19        CCTGTGCCTCCTTAACTACAGCATGGTGGGGGAGAAGTGTATTTTAAACGAACAGAATTCTTGCGCGGTGAAAAACGGGG
(THA) TS04_PvMSP1P-19        CCTGTGCCTCCTTAACTACAGCATGGTGGGGGAGAAGTGCATTTTAAACGAACAGAATTCTTGCGCGGTGAAAAACGGGG
(THA) TS05_PvMSP1P-19        CCTGTGCCTCCTTAACTACAGCATGGTGGGGGAGAAGTGCATTTTAAACGAACAGAATTCTTGCGCGGTGAAAAACGGGG
(THA) TS06_PvMSP1P-19        CCTGTGCCTCCTTAACTACAGCATGGTGGGGGAGAAGTGCATTTTAAACGAACAGAATTCTTGCGCGGTGAAAAACGGGG
```

```
                               ---------+---------+---------+---------+---------+---------+---------+---------+
                                       170       180       190       200       210       220       230       240
                               ---------+---------+---------+---------+---------+---------+---------+---------+
(PVX_099975) PvMSP1P-19        GGTGCGATTTGAAGGCAACGTGCGAGTTGAAAAAAAATCGCGTAAATTGCATTTGCCCCAAGGGCACGAAACCCATGCAC
(KOR) 201401_PvMSP1P-19        GGTGCGATTTGAAGGCAACGTGCGAGTTGAAAAAAAATCGCGTAAATTGCATTTGCCCCAAGGGCACGAAACCCATGCAC
(KOR) 201402_PvMSP1P-19        GGTGCGATTTGAAGGCAACGTGCGAGTTGAAAAAAAATCGCGTAAATTGCATTTGCCCCAAGGGCACGAAACCCATGCAC
(KOR) 201404_PvMSP1P-19        GGTGCGATTTGAAGGCAACGTGCGAGTTGAAAAAAAATCGCGTAAATTGCATTTGCCCCAAGGGCACGAAACCCATGCAC
(KOR) 201502_PvMSP1P-19        GGTGCGATTTGAAGGCAACGTGCGAGTTGAAAAAAAATCGCGTAAATTGCATTTGCCCCAAGGGCACGAAACCCATGCAC
(KOR) 201503_PvMSP1P-19        GGTGCGATTTGAAGGCAACGTGCGAGTTGAAAAAAAATCGCGTAAATTGCATTTGCCCCAAGGGCACGAAACCCATGCAC
(KOR) 201504_PvMSP1P-19        GGTGCGATTTGAAGGCAACGTGCGAGTTGAAAAAAAATCGCGTAAATTGCATTTGCCCCAAGGGCACGAAACCCATGCAC
(KOR) 201505_PvMSP1P-19        GGTGCGATTTGAAGGCAACGTGCGAGTTGAAAAAAAATCGCGTAAATTGCATTTGCCCCAAGGGCACGAAACCCATGCAC
(KOR) 201602_PvMSP1P-19        GGTGCGATTTGAAGGCAACGTGCGAGTTGAAAAAAAATCGCGTAAATTGCATTTGCCCCAAGGGCACGAAACCCATGCAC
(KOR) 201603_PvMSP1P-19        GGTGCGATTTGAAGGCAACGTGCGAGTTGAAAAAAAATCGCGTAAATTGCATTTGCCCCAAGGGCACGAAACCCATGCAC
(KOR) 201604_PvMSP1P-19        GGTGCGATTTGAAGGCAACGTGCGAGTTGAAAAAAAATCGCGTAAATTGCATTTGCCCCAAGGGCACGAAACCCATGCAC
(KOR) 201605_PvMSP1P-19        GGTGCGATTTGAAGGCAACGTGCGAGTTGAAAAAAAATCGCGTAAATTGCATTTGCCCCAAGGGCACGAAACCCATGCAC
(MYA) KV01_PvMSP1P-19          GGTGCGATTTGAAGGCAACGTGCGAGTTGAAAAAAAATCGCGTAAATTGCATTTGCCCCAAGGGCACGAAACCCATGCAC
(MYA) KV02_PvMSP1P-19          GGTGCGATTTGAAGGCAACGTGCGAGTTGAAAAAAAATCGCGTAAATTGCATTTGCCCCAAGGGCACGAAACCCATGCAC
(MYA) KV03_PvMSP1P-19          GGTGCGATTTGAAGGCAACGTGCGAGTTGAAAAAAAATCGCGTAAATTGCATTTGCCCCAAGGGCACGAAACCCATGCAC
(MYA) KV04_PvMSP1P-19          GGTGCGATTTGAAGGCAACGTGCGAGTTGAAAAAAAATCGCGTAAATTGCATTTGCCCCAAGGGCACGAAACCCATGCAC
(MYA) KV07_PvMSP1P-19          GGTGCGATTTGAAGGCAACGTGCGAGTTGAAAAAAAATCGCGTAAATTGCATTTGCCCCAAGGGCACGAAACCCATGCAC
(MYA) KV08_PvMSP1P-19          GGTGCGATTTGAAGGCAACGTGCGAGTTGAAAAAAAATCGCGTAAATTGCATTTGCCCCAAGGGCACGAAACCCATGCAC
(MYA) KV09_PvMSP1P-19          GGTGCGATTTGAAGGCAACGTGCGAGTTGAAAAAAAATCGCGTAAATTGCATTTGCCCCAAGGGCACGAAACCCATGCAC
(MYA) KV10_PvMSP1P-19          GGTGCGATTTGAAGGCAACGTGCGAGTTGAAAAAAAATCGCGTAAATTGCATTTGCCCCAAGGGCACGAAACCCATGCAC
(MYA) KV13_PvMSP1P-19          GGTGCGATTTGAAGGCAACGTGCGAGTTGAAAAAAAATCGCGTAAATTGCATTTGCCCCAAGGGCACGAAACCCATGCAC
(MYA) KV14_PvMSP1P-19          GGTGCGATTTGAAGGCAACGTGCGAGTTGAAAAAAAATCGCGTAAATTGCATTTGCCCCAAGGGCACGAAACCCATGCAC
(MYA) KV15_PvMSP1P-19          GGTGCGATTTGAAGGCAACGTGCGAGTTGAAAAAAAATCGCGTAAATTGCATTTGCCCCAAGGGCACGAAACCCATGCAC
(MYA) KV16_PvMSP1P-19          GGTGCGATTTGAAGGCAACGTGCGAGTTGAAAAAAAATCGCGTAAATTGCATTTGCCCCAAGGGCACGAAACCCATGCAC
(THA) LS02_PvMSP1P-19          GGTGCGATTTGAAGGCAACGTGCGAGTTGAAAAAAAATCGCGTAAATTGCATTTGCCCCAAGGGCACGAAACCCATGCAC
(THA) PR01_PvMSP1P-19          GGTGCGATTTGAAGGCAACGTGCGAGTTGAAAAAAAATCGCGTAAATTGCATTTGCCCCAAGGGCACGAAACCCATGCAC
(THA) TS02_PvMSP1P-19          GGTGCGATTTGAAGGCAACGTGCGAGTTGAAAAAAAATCGCGTAAATTGCATTTGCCCCAAGGGCACGAAACCCATGCAC
(THA) TS03_PvMSP1P-19          GGTGCGATTTGAAGGCAACGTGCGAGTTGAAAAAAAATCGCGTAAATTGCATTTGCCCCAAGGGCACGAAACCCATGCAC
(THA) TS04_PvMSP1P-19          GGTGCGATTTGAAGGCAACGTGCGAGTTGAAAAAAAATCGCGTAAATTGCATTTGCCCCAAGGGCACGAAACCCATGCAC
(THA) TS05_PvMSP1P-19          GGTGCGATTTGAAGGCAACGTGCGAGTTGAAAAAAAATCGCGTAAATTGCATTTGCCCCAAGGGCACGAAACCCATGCAC
(THA) TS06_PvMSP1P-19          GGTGCGATTTGAAGGCAACGTGCGAGTTGAAAAAAAATCGCGTAAATTGCATTTGCCCCAAGGGCACGAAACCCATGCAC
```

	---------+--------
	250
	---------+--------
(PVX_099975) PvMSP1P-19	GAGGGGGTCGTGTGCAGC
(KOR) 201401_PvMSP1P-19	GAGGGGGTCGTGTGCAGC
(KOR) 201402_PvMSP1P-19	GAGGGGGTCGTGTGCAGC
(KOR) 201404_PvMSP1P-19	GAGGGGGTCGTGTGCAGC
(KOR) 201502_PvMSP1P-19	GAGGGGGTCGTGTGCAGC
(KOR) 201503_PvMSP1P-19	GAGGGGGTCGTGTGCAGC
(KOR) 201504_PvMSP1P-19	GAGGGGGTCGTGTGCAGC
(KOR) 201505_PvMSP1P-19	GAGGGGGTCGTGTGCAGC
(KOR) 201602_PvMSP1P-19	GAGGGGGTCGTGTGCAGC
(KOR) 201603_PvMSP1P-19	GAGGGGGTCGTGTGCAGC
(KOR) 201604_PvMSP1P-19	GAGGGGGTCGTGTGCAGC
(KOR) 201605_PvMSP1P-19	GAGGGGGTCGTGTGCAGC
(MYA) KV01_PvMSP1P-19	GAGGGGGTCGTGTGCAGC
(MYA) KV02_PvMSP1P-19	GAGGGGGTCGTGTGCAGC
(MYA) KV03_PvMSP1P-19	GAGGGGGTCGTGTGCAGC
(MYA) KV04_PvMSP1P-19	GAGGGGGTCGTGTGCAGC
(MYA) KV07_PvMSP1P-19	GAGGGGGTCGTGTGCAGC
(MYA) KV08_PvMSP1P-19	GAGGGGGTCGTGTGCAGC
(MYA) KV09_PvMSP1P-19	GAGGGGGTCGTGTGCAGC
(MYA) KV10_PvMSP1P-19	GAGGGGGTCGTGTGCAGC
(MYA) KV13_PvMSP1P-19	GAGGGGGTCGTGTGCAGC
(MYA) KV14_PvMSP1P-19	GAGGGGGTCGTGTGCAGC
(MYA) KV15_PvMSP1P-19	GAGGGGGTCGTGTGCAGC
(MYA) KV16_PvMSP1P-19	GAGGGGGTCGTGTGCAGC
(THA) LS02_PvMSP1P-19	GAGGGGGTCGTGTGCAGC
(THA) PR01_PvMSP1P-19	GAGGGGGTCGTGTGCAGC
(THA) TS02_PvMSP1P-19	GAGGGGGTCGTGTGCAGC
(THA) TS03_PvMSP1P-19	GAGGGGGTCGTGTGCAGC
(THA) TS04_PvMSP1P-19	GAGGGGGTCGTGTGCAGC
(THA) TS05_PvMSP1P-19	GAGGGGGTCGTGTGCAGC
(THA) TS06_PvMSP1P-19	GAGGGGGTCGTGTGCAGC
